# Hematological biomarkers of troponin, neutrophil-to-lymphocyte ratio, and monocyte-to-lymphocyte ratio serve as effective predictive indicators of high-risk mortality in acute coronary syndrome

**DOI:** 10.37796/2211-8039.1425

**Published:** 2023-12-01

**Authors:** Bryan G. de Liyis, Angela F. Ciaves, Marwa H. Intizam, Pierre J. Jusuf, I. Made J. Rina Artha

**Affiliations:** a Faculty of Medicine, Universitas Udayana, Denpasar, Bali, Indonesia; b Department of Cardiology and Vascular Medicine, Universitas Udayana, Denpasar, Bali, Indonesia

**Keywords:** Acute coronary syndrome, Monocyte to lymphocyte ratio, Mortality, Neutrophil to lymphocyte ratio, Troponin

## Abstract

**Background:**

Assessing high-risk mortality in acute coronary syndrome (ACS) patients, encompassing ST-Elevation Myocardial Infarction (STEMI), Non-ST-Elevation Myocardial Infarction (NSTEMI), and Unstable Angina Pectoris (UAP), is crucial. However, the prognostic significance of hematological parameters in predicting high-risk mortality in ACS patients remains uncertain despite advancements in ACS research.

**Aim:**

The aim was to investigate prognostic significance of hematological parameters troponin, Creatine Kinase-MB (CKMB), Neutrophil-to-Lymphocyte Ratio (NLR), Platelet-to-Lymphocyte Ratio (PLR), Monocyte-to-Lymphocyte Ratio (MLR), Basophil-to-Lymphocyte Ratio (BLR), and Eosinophil-to-Lymphocyte Ratio (ELR) levels in predicting high-risk mortality in ACS patients.

**Methods:**

In this retrospective observational study, data from medical records of 115 patients with ACS, including 40 with STEMI, 38 with NSTEMI, and 37 with UAP, were analyzed. Patients were selected using stratified random sampling, whereby five patients were randomly chosen each month from January 2021 to December 2022 while maintaining a 1:1:1 ratio of selection.

**Results:**

Troponin (r = 0.519) and NLR (r = 0.484) showed moderate positive correlations with high-risk STEMI mortality. Meanwhile, troponin (r = 0.387), NLR (r = 0.279), PLR (r = 0.276), MLR (r = 0.250), BLR (r = 0.237), and ELR (r = −0.344) were found to be significantly correlated with high-risk ACS mortality. Troponin, CKMB, NLR, and MLR were significant (AUC>0.7) for high-risk STEMI mortality, and Troponin, NLR, and MLR were significant for high-risk ACS mortality. The results of the multivariate regression analysis indicated that only Troponin (OR:2.049; 95%CI: 1.802–8.218; p = 0.014), NLR (OR:1.652; 95%CI: 1.306–7.753; p = 0.030), and MLR (OR:4.067; 95%CI: 1.182–13.987; p = 0.026) were capable of predicting high-risk ACS mortality. Sub-group analysis showed an increased risk of ACS mortality by GRACE score >140 in patients with elevated levels of Troponin (OR:2.787; 95%CI: 1.032–7.524; p < 0.05), NLR (OR:3.287; 95%CI: 1.340–8.059; p < 0.05), and MLR (OR:4.156; 95%CI: 1.634–10.569; p < 0.05) above the cut-off value.

**Conclusion:**

Troponin, NLR, and MLR levels above the cutoff independently predict high-risk mortality in ACS.

## 1. Introduction

Myocardial infarction, also referred to as a heart attack is a disease which occurs when there is a decrease in blood supply to the heart muscle and is mostly caused by coronary heart disease or CHD [1]. Acute myocardial infarction can be classified into two groups according to the presence or absence of ST wave elevation on the results electrocardiogram, myocardial infarction with ST elevation or STEMI and myocardial infarction without ST elevation or NSTEMI [2]. Myocardial infarction is a manifestation of ischemic heart disease, the main cause of death and morbidity which has an incidence of 1655 people for every 100,000 population and results in 7.4 million deaths each year, the number of which is expected to continue to increase [3,4]. This disease is more common in men and in the elderly with an average age of 68 years. Cases of myocardial infarction of the NSTEMI type are more common than STEMI and have a ratio of approximately 2.3:1 [5,6].

As previously mentioned, males and elders are groups that have a higher risk of myocardial infarction. There are also several other risk factors, such as a history of CHD from the patient or the patient’s family, diabetes, and disruption in kidney function [6]. Currently, myocardial infarction can be diagnosed when two of these conditions are met: patients showing ischemic symptoms, abnormalities in the ST segment or impulses to the left side of the heart being delayed, abnormalities in the Q wave, abnormalities in the motion of the heart wall that can be found on radiological examination of the heart, autopsy or angiography examination shows the presence of a thrombus. Patients with myocardial infarction have a high risk of death and almost half of patients who have received treatment must return to get treatment at the hospital in under one year. Therapy and early diagnosis, especially 30 min since the patient came to the hospital or within 6 h of onset can improve the patient’s prognosis [7].

The current method used for the diagnosis of myocardial infarction has several drawbacks which can extend the patient’s diagnosis time and cannot determine a clear prognosis. Not all patients with myocardial infarction have ischemic symptoms, and these patients require other tests such as an ECG examination to check for abnormalities in heart waves that can lead to a diagnosis of myocardial infarction. Although very specific, the sensitivity of the ECG to myocardial infarction is only about 30% [8,9]. It was some of the matters mentioned above that motivated the authors to conduct this research in the hope of finding a method of diagnosis and determining a prognosis that is easier, more accurate, and more cost-efficient in order to help treat patients with myocardial infarction furthermore.

The use of these biomarkers may help clinicians to more accurately stratify patients based on their risk of mortality and provide appropriate and timely management. Overall, this research has the potential to improve the clinical management and outcomes of patients with ACS, making it an important area of investigation.

## 2. Materials and methods

### 2.1. Study design and study participants

This retrospective observational study was conducted at a single center and approved by the Ethics Committee of Sanglah General Hospital, Bali, Indonesia, which is affiliated with Udayana University. Data for the study was obtained from medical records of 115 patients admitted to the hospital, including 40 with STEMI, 38 with NSTEMI, and 37 with UAP. Patients were selected using stratified random sampling, with 5 patients selected randomly each month from January 2021 to December 2022, while maintaining an appropriate ratio of selection (1:1:1) to prevent selection bias. STEMI was diagnosed by the presence of ST-segment elevation on ECG and elevated cardiac biomarkers, while NSTEMI was diagnosed by the absence of ST-segment elevation on ECG and elevated cardiac biomarkers. UAP was diagnosed in the absence of ST-segment elevation on ECG and normal cardiac biomarkers. If the cardiac biomarker was elevated from a previously tested normal cardiac marker in a patient diagnosed with UAP on the same day, the number of the elevated cardiac biomarkers was recorded. Inclusion criteria for the study were age greater than 18 years and a diagnosis of STEMI, NSTEMI, or UAP. Exclusion criteria included severe diseases with a life expectancy of less than half a year, such as late malignant tumors, autoimmune diseases, and infections, as well as pregnant or lactating women.

### 2.2. Data collection and imputation

Prior to treatment initiation, data entry was conducted based on predetermined variables. An electronic medical record system was used to extract demographic information, vital signs, clinical characteristics, laboratory test results, and outcomes of patients upon admission and throughout their hospitalization. The initial laboratory test results after admission, including blood tests for routine examination, cardiac biomarkers, cholesterol, blood glucose, biochemical test for liver and kidney function, and blood coagulation function, were collected. NLR, PLR, MLR, BLR, and ELR were derived from the absolute count of neutrophil, platelet, monocyte, basophil, and eosinophil each divided by the absolute count of lymphocyte. The Killip class was assigned based on clinical characteristics. Killip class I indicate a lack of observable clinical heart failure symptoms, while Killip class II denotes individuals with physical manifestations of rales or crackles in the lungs, an S3 heart sound, and raised jugular venous pressure. Killip class III is assigned to individuals with acute pulmonary edema, and Killip class IV is assigned to those who exhibit cardiogenic shock or hypotension. Grace score was computed using the GRACE 2.0 ACS risk calculator by dr. Joel Gore. High-risk mortality was calculated with a GRACE score of ≥140. The outcome was determined by all-cause mortality during hospitalization. Experienced physicians and statisticians reviewed and verified the data. Missing data were imputed using SPSS Ver.25 software through regression imputation after excluding variables with excessive missing values (>50%). The reliability of the imputed data was evaluated by comparing the baseline data before and after imputation.

### 2.3. Study outcomes

The main objective of the study was to determine the prognostic value of cardiac biomarkers, including Troponin and CKMB, as well as NLR, PLR, MLR, BLR and ELR, for predicting high-risk mortality in ACS patients based on the GRACE score. The study evaluated the odds ratio and 95% confidence interval for the cut-off value that was found to be associated with high-risk mortality. In addition, the secondary objective was to compare the outcomes of two sub-groups based on the cut-off values with respect to admission mortality and other parameters. The study further calculated correlations, the area under the curve (AUC), cut-off values, sensitivity, and specificity for each diagnosis for all the patients included in the analysis.

### 2.4. Ethical approval

The study was conducted in accordance with the International Conference on Harmonization – Good Clinical Practice (ICH-GCP) and approved by the Research Ethics Committee Faculty of Medicine Universitas Udayana, Bali, Indonesia (Protocol Number: 2022.01.1.0317 on 11 March 2022, Research ID 220606.249 on 13 July 2022). All patients enrolled in the study gave their informed written consent to be included in the present analysis.

### 2.5. Statistical analysis

In this study, the data analysis was performed using the Statistical Package for the Social Sciences (SPSS) version 25 (SPSS Inc., Chicago, IL, USA). To determine the normality of continuous variable distribution, the Kolmogorov–Smirnov test (KS-test) was employed. The results were presented as mean ± standard deviation, ratio, 95% IC, cutoff value, sensitivity, and specificity. The relationship between the stage and the Troponin, CKMB, NLR, PLR, MLR, BLR, and ELR was evaluated by utilizing the Mann–Whitney test. Categorical data were analyzed by chi-square. The cutoff values of these variables were determined using ROC analysis with Area under the Curve (AUC), and the sensitivity and specificity for each variable and risk model were determined using multivariate analysis levels. For analyzing the cut-off value of NLR and comparing the efficiencies of different ratios, the ROC curve was used. The data were reported as mean ± standard deviation, ratio, 95% IC, cutoff value, sensitivity, and specificity. The cutoff point for the ratio was determined using the receiver operating characteristic (ROC) curve by considering the value of sensitivity and specificity. The cutoff values were utilized to establish the sensitivity and specificity of each variable and risk model at the multivariate analysis level. The statistical significance level was set at p ≤ 0.05.

## 3. Results

### 3.1. Baseline characteristic of the subjects

The baseline characteristics of the study are shown in Table 1. A total of 115 patients were enrolled in this retrospective study; 40 patients were diagnosed with STEMI, 38 with NSTEMI and 37 with UAP. There was no significant difference in gender, age, heart rate, blood pressure mortality or Killip class between patients with STEMI, NSTEMI, and UAP. However, a significant difference was found between groups for GRACE scores; with STEMI (134.70 ± 41.699) having the highest score followed by NSTEMI (111.45 ± 36.976) and UAP (94.11 ± 35.499). In terms of blood parameters, only Troponin (p < 0.001), WBC (p = 0.002), number of neutrophils (p = 0.042), number of eosinophils(p=0.040), and ELR (p=0.023) were found to be significantly different between the groups.

### 3.2. Correlation of hematology parameters with high-risk mortality by GRACE score in STEMI, NSTEMI, UAP, and ACS

Bivariate correlation analysis and partial correlation analysis were conducted on blood parameters, such as Troponin, CKMB, NLR, PLR, BLR, MLR, and ELR, towards GRACE Score in the context of STEMI, NSTEMI, UAP, and ACS (Table 2). The partial correlation analysis controlled for factors such as age, gender, Killip class, mean arterial pressure, and mortality. Without adjustments, Troponin (r = 0.382), NLR (r = 0.387), and MLR (r = 0.381) showed slight positive correlations with high-risk STEMI mortality, whereas ELR (r = −0.399) showed a slight negative correlation with highrisk STEMI mortality. Similarly, Troponin (r = 0.465) and NLR (r = 0.328) showed a moderate positive correlation and slight positive correlation with high-risk ACS mortality respectively, whereas ELR (r = −0.297) showed a slight negative correlation with high-risk ACS mortality. MLR (r = 0.217) was found to have a low positive correlation with high-risk ACS mortality.

Following adjustments, both Troponin (r = 0.519) and PLR (r = 0.484) showed moderate correlations with high-risk STEMI mortality. In terms of UAP, partial correlation analysis found that NLR (r = 0.527), PLR (r = 0.558), MLR (r = 0.473), and BLR (r = 0.445) showed moderate positive correlations with high-risk UAP mortality. Moreover, Troponin and all lymphocytes–ratios parameters were found to be significantly correlated with high-risk ACS mortality. Troponin (r = 0.387) showed a slight positive correlation with high-risk ACS mortality, whereas NLR (r = 0.279), PLR (r = 276), MLR (r = 0.250), and BLR (r = 0.237) showed low positive correlations with high-risk ACS mortality. Conversely, ELR (r=−0.344) had the opposite effect.

### 3.3. ROC curve of sensitivity and specificity of troponin, CKMB, NLR, PLR, MLR, BLR, and ELR as hematological parameters in the high-risk mortality

**Fig. 1:**
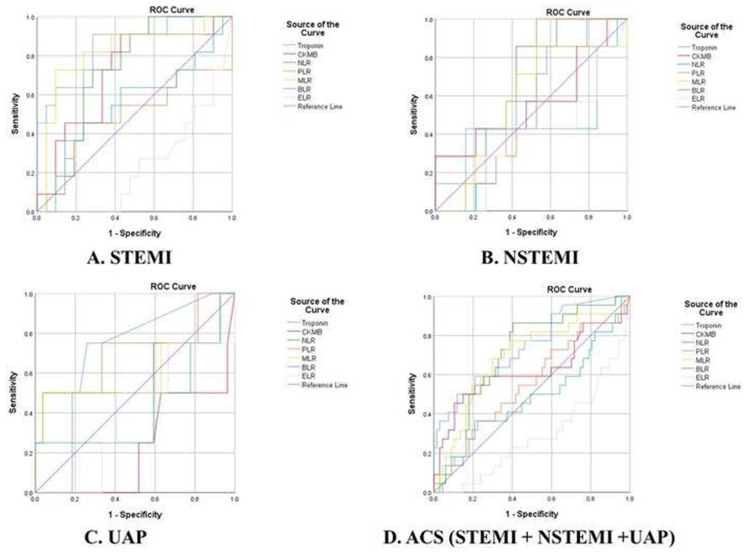
Receiver Operating Characteristic (ROC) analysis of blood parameters, including Troponin, CKMB, NLR, PLR, BLR, MLR, and ELR, as predictors of high-risk mortality as assessed by the GRACE Score. The diagonal reference line, which represents a null value, serves as a comparison to the area under the curve (AUC) of the variables in question. (A) Troponin, CKMB, NLR, and MLR were the only variables that displayed a significant AUC value greater than 0.70, suggesting their diagnostic value as predictors of high-risk STEMI mortality. (B, C) None of the parameter showed significant results in regards to NSTEMI and UAP. (D) Troponin, NLR, and MLR were the only variables that displayed a significant AUC value greater than 0.70, suggesting their diagnostic value as predictors of high-risk ACS mortality.

The ability of various biomarkers, including Troponin, CKMB, NLR, PLR, MLR, BLR, and ELR, to predict high-risk mortality in the context of GRACE Score was investigated using the receiver operating characteristic (ROC) curve analysis (Fig. 1). With regards to STEMI, the study findings revealed that only Troponin, CKMB, NLR, and MLR had significant and adequate diagnostic value, as evidenced by their respective AUC values exceeding 0.7. Further analysis of Troponin, NLR, and MLR as predictive parameters for mortality in high-risk STEMI patients showed that Troponin had a sensitivity of 72.7% and specificity of 71.4% at a cut-off value of 5193.6, while CKMB exhibited a sensitivity of 63.6% and specificity of 66.7% at a cut-off value of 116.05. Likewise, NLR had a sensitivity of 72.7% and specificity of 71.4% at a cut-off value of 8.13, and MLR showed a sensitivity of 72.7% and specificity of 76.2% at a cut-off value of 0.4735.

In terms of ACS, the results indicated that only Troponin, NLR, and MLR had significant sufficient diagnostic value as evidenced by their respective area under the curve (AUC) values exceeding 0.7. Further evaluation of Troponin, NLR, and MLR as predictive parameters in high-risk ACS mortality revealed that Troponin had a sensitivity of 63.6% and specificity of 64.2% at a cut-off value of 234.2, NLR demonstrated a sensitivity of 63.6% and specificity of 67.2% at a cut-off value of 6.23, and MLR had a sensitivity of 68.2% and specificity of 68.7% at a cut-off value of 0.4144 (Table 3).

### 3.4. Risk analysis model of hematological parameters as a predictive value in the high-risk ACS mortality

A two-step risk analysis was conducted utilizing crosstabulation and multinomial regression, incorporating the cutoff values obtained from the ROC analysis (Fig. 1D). The bivariate risk analysis indicated that Troponin, NLR, MLR, and ELR were significantly associated with an increased risk of ACS mortality, with MLR exhibiting the highest odds ratio value. However, the results of the multivariate regression analysis indicated that only Troponin (OR: 2.049; 95%CI: 1.802–8.218; p = 0.014), NLR (OR: 1.652; 95%CI: 1.306–7.753; p = 0.030), and MLR (OR: 4.156; 95%CI: 1.634–10.569; p = 0.002) were capable of predicting high-risk ACS mortality, while ELR was found to be insignificant (Table 4).

### 3.5. ACS mortality risk outcomes of the two sub-groups evaluated separately according to the optimal cut-off value of troponin, NLR, and MLR

The investigation involved stratifying the ACS patient cohort into two distinct groups based on the determined optimal cut-off values from ROC curve analysis, namely 234.2000 for Troponin, 6.2256 for NLR, and 0.4144 for MLR as indicated in Table 5. To further dissect the spectrum of ACS outcomes, the risk of mortality was evaluated using a GRACE score threshold of 140. Elevated Troponin levels exceeding 234.2 were associated with a notable 2.79-fold increase in the risk of ACS mortality (OR: 2.787; 95% CI: 1.032–7.524). Similarly, NLR levels surpassing 6.22 demonstrated a 3.29-fold amplified risk of ACS mortality (OR: 3.287; 95% CI: 1.340–8.059), while elevated MLR values exceeding 0.42 were linked to a substantial 4.16-fold escalated risk of ACS mortality (OR: 4.156; 95% CI: 1.634–10.569). These findings collectively underscore the profound prognostic potential of these biomarkers in forecasting adverse outcomes within the ACS patient population.

## 4. Discussion

The analysis of several essential attributes of this study showed statistically significant variances between groups of STEMI, NSTEMI and UAP. The mean GRACE score, Troponin level, and WBC count was found to be highest in STEMI, followed by NSTEMI then UAP. Most elevated neutrophil count was found in the NSTEMI group, meanwhile eosinophil level and eosinophil-tolymphocyte ratio (ELR) was highest in the UAP group. The percentage of basic characteristics examined in the study such as sex and age were in accordance with several other studies. It was shown in this study that ACS patients are predominated by the male sex, mostly in STEMI with the percentage of 77,5% This reflects a study by Ginanjar et al. which displayed 76.2% of the STEMI group are male patients with the median age of 57 years old [10]. Another similar result from Wojcik et al. showing 92% of the ACS patients are male and the median age was 70 years old [11].

This study assessed the patients’ GRACE score as the clinical risk predictor for mortality and morbidity of the diseases. The GRACE score has long been recommended as a risk stratifying tool and is not restricted to any ST-segment alterations [12]. The result of this study affirmed considerable difference between the groups for GRACE score, which was shown highest in STEMI patients (134.70 ± 41.699) then followed by NSTEMI (111.45 ± 36.976) and UAP (94.11 ± 35.499). This does not show much resemblance with a study from Abuassi et al. which showed higher GRACE score in NSTEMI rather than those in STEMI. The GRACE score result was in line with increased mortality. It was also informed in their study that by 6 months after their discharge from the hospital, 3.7% patients from the STEMI group and 4.8% from the NSTEMI had died [13]. This difference may be fractionally due to the different distribution among several prognostic variables and geographical variance affecting the condition of the populations.

Another important characteristic analyzed in this study is Troponin as one of the cardiac biomarkers, whose elevation is caused by ischemic process. The Troponin, commonly Troponin I has not been used as a regular or routine examination in Indonesia as it is also not widely available and accessible in the health facilities throughout Indonesia. A related study by Kurniawan et al. showed similar result for Troponin level in different groups as the one in this study. The Troponin value in the STEMI group is higher than the NSTEMI patient group [14]. This difference in the Troponin level between STEMI and NSTEMI group may be due to the pathophysiology of each STEMI and NSTEMI. The thrombus blocks the whole lumen of the artery in the case of STEMI, meanwhile it only partially blocks the lumen in NSTEMI. The total occlusion in STEMI is due to thrombus, usually formed from atherosclerotic plaque rupture. This complete occlusion will produce greater decrease of oxygen supply to the cardiac muscle cells compared to NSTEMI case. The diminishing of oxygen supply later will result in ionic and osmotic imbalance of the cells leading to necrosis which will disrupt the membrane of cardiomyocytes, resulting in proteolytic degradation of the Troponin-tropomyosin protein complex which is the main trigger for Troponin release [14]. Another study by Buber et al. also supports the same assumption, on 175 patients with STEMI, peak levels of high-sensitivity cardiac Troponin I at 8 h were independent predictors of all-cause mortality, myocardial infarction and in-hospital failure. The patients who has higher peak Troponin level in the study also died at 30 days and 1 year following the hospitalization [15].

WBC count in this study was found to be highest in STEMI group meanwhile the neutrophil level was most increased in NSTEMI patients. Such finding does not show similarity in a study by Gunes et al. where both WBC and neutrophil count was highest in STEMI patients. It is also stated that increased WBC count goes in line with increased neutrophil count in most cases of ACS [16]. The widely trusted predictive marker for ACS is NLR, whose both sensitivity and specificity are above 60%. A study by Yilmaz et al. stated that there was an increased incidence of NSTEMI in patients with high NLR, it is also an independent predictors of coronary thrombus formation in NSTEMI patients along with neutrophil and lymphocyte count [17].

After adjusting other factors, correlations with high-risk mortality by GRACE Score are found in STEMI for Troponin and PLR. In adjusted UAP group, correlations are found for NLR, PLR, MLR, and BLR. Overall, there are correlations of ACS with Troponin, NLR, PLR, MLR, BLR, and ELR for high-risk mortality after age, gender, Killip class, mean arterial pressure, and mortality are adjusted. However, there are no correlations found in adjusted NSTEMI group. Troponin level in STEMI have moderate correlation (p = 0.519) with high-risk mortality by GRACE Score, which is supported by previous research which stating that higher peak level of Troponin is associated with higher mortality of STEMI patients within 30 days [18]. Another blood parameter that has moderate correlation with high-risk mortality in STEMI is PLR (p = 0.484). There is a study by Dong, Guoxia et all in 2021 finding that PLR are associated with STEMI outcome and can predict the mortality of STEMI patient [19].

In the UAP group NLR has slight positive correlation (p = 0.527) with high-risk mortality by GRACE Score after adjustment. It is stated in another research that NLR can determine the mortality caused by cardiovascular disease in UAP patients [20]. PLR, MLR, BLR is also moderately positively correlated with high-risk mortality in adjusted UAP. Generally, Troponin, NLR, PLR, MLR, and BLR have weak positive association with high-risk mortality by GRACE Score in adjusted ACS. Troponin level (p = 0.387) is also found to be related positively to ACS mortality in a cohort study done by Kaura, Amit et all in 2019 [21].

This study is focused on examining the predictive efficacy of complete blood count (CBC) ratios (such as NLR, PLR, BLR, MLR, and ELR) in ACS in this analysis of patients from their medical records. The study mainly discovered that along with Troponin, NLR and MLR were greater predictors of high-risk ACS mortality than other prevalent CBC ratios and some of the cardiac biomarkers. The predictive role of NLR and MLR on the mortality of high-risk ACS patients has been demonstrated in several published papers. The use of hematologic parameter in determining high-risk ACS mortality has become more common among clinicians. Many researches have examined the connection between NLR and ACS, AMI in particular. NLR could predict unfavorable outcomes in ACS according to earlier studies with small sample sizes [22]. The potential for NLR to predict major adverse cardiovascular events (MACE) following ACS was examined in a retrospective study involving 107 patients, and it was discovered that a high NLR (6.07) was a significant predictor for the poorest outcome [23]. Dong et al. conducted a study that included 9406 patients and reported the connection between high NLR values and high mortality rates. The NLR value of 5.0 might be the cut-off value for ACS risk, whilst in this study the cut-off value is 6.23 [24]. Regarding the predictive role of MLR, a study by Li et al. reported NLR and MLR to be significantly and independently associated with major adverse cardiovascular events (MACE), including all-cause death, non-fatal ischemic stroke, and non-fatal myocardial infarction [25]. According to another study, MLR was independently linked to ACS and might be used to gauge the degree of coronary lesions [26]. The findings of Song et al. investigation’s yielded similar results [27]. Monocytes, one of the most significant inflammatory cells, play a direct role in the emergence and progression of atherosclerosis. As monocytes consume oxidized lipoprotein, which can trigger a number of inflammatory signaling molecules and oxidized free radicals in plaque, they differentiate into macrophages and stick to vascular endothelium [28,29].

An increased risk of mortality due to ACS was also observed in patients with elevated levels of Troponin (OR: 2.787; 95%CI: 1.032–7.524), NLR (OR: 3.287; 95% CI: 1.340–8.059), and MLR (OR: 4.156; 95%CI: 1.634–10.569) based on the stratification of GRACE score and this study suggested that NLR and MLR were found to be related to the severity of ACS. These findings are in line with several other studies that also assess the predictive value of CBC ratios. For example, Ji et al. found a significant relationship between NLR with in-hospital death in ACS patients [30]. In addition, it is also observed that elevated levels of Troponin (OR: 2719; 95%CI: 1.140–6.482), NLR (OR: 5.039; 96%CI: 2.113–12.020), andMLR (OR: 2.255; 95%CI: 1.037–4.902) were associated with a higher level of WBC in ACS patients. Theoretically, Neutrophils and lymphocytes play a well-known proinflammatory role in immune system regulation [31,32]. Increases in lymphocyte apoptosis, an increased risk of infection, adverse cardiovascular events, and enhanced lymphocyte apoptosis have all been linked to systemic inflammation [33,34]. Increased neutrophil counts and decreased lymphocyte numbers are the foundation for elevated NLR values, which represent the delicate balance between systemic inflammation and immunological response. In addition to their established hemostatic role, platelets play a vital part in the inflammatory process and immunological responses [35–37]. Measures of acute myeloid-driven innate immune responses, such as NLR and MLR, are reported to chronic lymphocyte-driven immunological memory, which is reflected by lymphocyte counts. An immunological imbalance between a probable continuous clinical or sub-clinical acute inflammation and a compromised immune response to pathogens may be reflected by elevated MLR and NLR. Atherosclerosis is formed and developed in part by inflammation, which has also been recognized as a major pathogenic component and damaging mediator of ischemia-reperfusion injury in ACS patients [38,39]. The prognosis of ACS patients might be impacted by inflammatory cells such white blood cells and inflammation-related indices likeMLRandNLR [40].

Foremost, a notable limitation pertains to the temporal dimension of mortality, which is a pivotal factor when assessing predictive indicator markers. The study, while extensively investigating the predictive capacities of various hematological biomarkers, does not delve into the intricate relationship between the timing of mortality events and the identified markers. Recognizing that mortality patterns can vary over time, this temporal dimension should be an avenue of future investigation, affording a more comprehensive understanding of the markers’ prognostic relevance. Moreover, a more comprehensive evaluation of cardiovascular-related deaths in terms of biomarker predictive values would be a pertinent augmentation to this study’s scope. Furthermore, while the study diligently examines the predictive capabilities of individual hematological biomarkers, the synergistic potential of a combinative assessment of multiple biomarkers remains an avenue for future inquiry.

## 5. Conclusions

The discovery and validation of these predictive biomarkers on the negative outcome of highrisk ACS is a critical point in improving their quality of life and increasing their life expectancy. According to the result of this study and the recent literature, the values of Troponin, NLR, and MLR are independent factors in predicting the negative outcome of high-risk ACS patients, which can end with mortality. The NLR and MLR showed a potential use for predicting the high-risk ACS mortality. Moreover, with NLR and MLR as a part of blood routine examination, a more accessible and costefficient alternative for predicting the prognosis of high-risk ACS becomes available. However, other inflammatory conditions and the limitation of this study may affect the result. Therefore, the use of these parameters warrants more comprehensive and prospective research study before further implementation in the clinical settings.

## Figures and Tables

**Table 1:** Basic characteristics and blood parameters of enrolling patients.

Characteristics	STEMI (n = 40)	NSTEMI (n = 38)	UAP (n = 37)	*p*	ACS (n = 115)
Male Sex (%)	31 (77.5)	27 (71.1)	26 (70.3)	0.737	84 (73.0)
Mean Age ± SD (years)	62.78 ± 13.392	62.50 ± 13.242	59.05 ± 13.002	0.398	61.49 ± 13.210
Mean Heart Rate ± SD (bpm)	78.16 ± 20.085	79.61 ± 14.702	74.39 ± 16.543	0.406	77.43 ± 17.302
Mean Systole ± SD (mmHg)	114.63 ± 17.315	118.44 ± 17.970	125.01 ± 26.917	0.098	119.23 ± 21.322
Mean Diastole ± SD (mmHg)	70.48 ± 10.260	73.62 ± 8.723	72.55 ± 13.332	0.434	72.18 ± 10.890
Mean Arterial Pressure ± SD (mmHg)	85.20 ± 11.863	88.56 ± 10.527	90.04 ± 16.211	0.250	87.87 ± 13.088
Mean GRACE Score ± SD	134.70 ± 41.699	111.45 ± 36.976	94.11 ± 35.499	<0.001*	113.96 ± 41.443
Mortality (%)	2 (5.0)	7 (18.4)	4 (10.8)	0.176	13 (11.3)
Killip Class (%)				0.091	
I	22 (55.0)	21 (55.3)	28 (75.7)		71 (61.7)
II	6 (15.0)	7 (18.4)	5 (13.5)		18 (15.7)
III	1 (2.5)	5 (13.2)	0 (0.0)		6 (5.2)
IV	11 (27.5)	5 (13.2)	4 (10.8)		20 (17.4)
		**Mean Blood Parameters** **±** **SD**			
Troponin (pg/mL)	22120.24 ± 41.699	1912.13 ± 6435.538	198.74 ± 912.735	<0.001*	8784.38 ± 25575.716
CKMB (U/L)	214.81 ± 272.478	3490.72 ± 18561.431	57.471 ± 151.575	0.343	1160.17 ± 10250.579
WBC (10^3^/μL)	13.46 ± 4.596	12.23 ± 4.338	9.97 ± 3.930	0.002*	11.93 ± 4.507
NE# (10^3^/μL)	10.95 ± 4.430	12.85 ± 15.483	7.31 ± 3.928	0.042*	10.41 ± 9.726
MO# (10^3^/μL)	0.81 ± 0.570	0.85 ± 0.514	0.68 ± 0.357	0.276	0.78 ± 0.493
EO# (10^3^/μL)	0.08 ± 0.119	0.18 ± 0.287	0.19 ± 0.212	0.040*	0.15 ± 0.221
BA# (10^3^/μL)	0.05 ± 0.091	0.05 ± 0.079	0.03 ± 0.027	0.459	0.04 ± 0.072
LY# (10^3^/μL)	1.66 ± 0.727	1.85 ± 0.957	1.75 ± 0.782	0.589	1.75 ± 0.829
PLT (10^3^/μL)	257.54 ± 107.332	253.87 ± 82.764	275.12 ± 108.803	0.623	262.09 ± 100.006
NLR	8.61 ± 7.364	10.834 ± 16.559	5.86 ± 6.325	0.154	8.45 ± 11.146
PLR	188.93 ± 145.881	183.95 ± 165.349	205.39 ± 199.678	0.851	192.58 ± 169.787
MLR	0.60 ± 0.654	0.58 ± 0.673	0.49 ± 0.443	0.706	0.56 ± 0.598
BLR	0.05 ± 0.119	0.04 ± 0.075	0.02 ± 0.042	0.532	0.04 ± 0.086
ELR	0.05 ± 0.064	0.09 ± 0.116	0.11 ± 0.111	0.023*	0.08 ± 0.101
RBC (10^6^/μL)	4.57 ± 0.842	4.53 ± 0.919	4.27 ± 0.944	0.294	4.46 ± 0.903
HGB (g/dL)	13.23 ± 2.157	13.09 ± 2.729	12.15 ± 2.407	0.118	12.84 ± 2.463
HCT (%)	39.08 ± 8.032	39.83 ± 8.704	36.69 ± 7.682	0.224	38.56 ± 8.189
MCV (fL)	89.09 ± 4.854	87.48 ± 9.797	86.46 ± 6.215	0.277	87.7 ± 7.253
MCH (pg)	29.11 ± 1.520	30.39 ± 9.237	28.717 ± 2.241	0.391	29.40 ± 5.485
MCHC (g/dL)	32.69 ± 1.093	32.61 ± 1.410	32.95 ± 2.496	0.681	32.75 ± 1.744
RDW (%)	12.85 ± 0.747	13.51 ± 1.557	13.33 ± 1.319	0.058	13.22 ± 1.267
MPV (fL)	10.02 ± 1.651	10.20 ± 1.539	9.61 ± 1.047	0.192	9.95 ± 1.452
PPT (s)	20.25 ± 28.545	13.10 ± 4.118	19.97 ± 45.559	0.529	17.82 ± 30.814
INR	1.46 ± 2.245	1.05 ± 0.214	1.81 ± 4.894	0.582	1.44 ± 3.071
APPT (s)	31.24 ± 7.967	28.61 ± 5.519	29.99 ± 12.275	0.491	30.01 ± 9.443
Total					
Cholesterol (mg/dL)	177.08 ± 39.993	189.88 ± 94.491	149.14 ± 53.861	0.274	173.14 ± 70.347
Triglyceride (mg/dL)	145.27 ± 74.579	149.11 ± 68.029	108.64 ± 62.423	0.229	134.77 ± 69.197
SGOT (U/L)	375.76 ± 1160.422	92.81 ± 197.269	27.68 ± 31.858	0.077	172.40 ± 711.250
SGPT (U/L)	274.82 ± 882.376	52.69 ± 62.964	27.79 ± 38.885	0.081	123.39 ± 534.453
Fasting Glucose (mg/dL)	186.75 ± 119.186	164.93 ± 100.839	119.39 ± 26.688	0.195	153.00 ± 88.446
HbA1C (%)	7.98 ± 3.078	7.97 ± 2.570	7.68 ± 1.715	0.973	7.91 ± 2.573
Creatine (mg/dL)	1.65 ± 1.405	2.10 ± 2.222	1.69 ± 1.704	0.493	1.81 ± 1.791
BUN (mg/dL)	22.11 ± 22.45	24.22 ± 18.805	22.45 ± 19.289	0.889	22.92 ± 20.022
Uric Acid (mg/dL)	7.33 ± 2.883	13.41 ± 20.534	6.35 ± 2.234	0.267	9.17 ± 12.584

SD, standard deviation; p, significance; STEMI, ST-elevation myocardial infarction; NSTEMI, non STelevation myocardial infarction; UAP, unstable angina pectoris; ACS, acute coronary syndrome; CKMB, creatinine kinase myocardial band; WBC, white blood cells; NE, neutrophil; MO, monocyte; EO, eosinophil; BA, basophil; LY, lymphocyte; PLT, platelet; NLR, neutrophil to lymphocyte ratio; PLR, platelet to lymphocyte ratio; MLR, monocyte to lymphocyte ratio; BLR, basophil to lymphocyte ratio; ELR, eosinophil to lymphocyte ratio; RBC, red blood cells; HGB, hemoglobin; HCT, hematocrit; MCV, mean corpuscular volume; MCH, mean corpuscular hemoglobin; MCHC, mean corpuscular hemoglobin concentration; RDW, red blood cell distribution width; MPV, mean platelet volume; PPT, parameter prothrombin time; INR, international normalized ratio; APPT, activated partial thromboplastin time; SGOT, serum glutamic oxaloacetic transaminase; SGPT, serum glutamic pyruvic transaminase; HbA1C, hemoglobin A1C; BUN, blood urea nitrogen.

**Table 2:** Correlation between blood parameters with GRACE score before and after adjustments for age, gender, Killip class, mean arterial pressure, and mortality.

State Variable	Parameter	r Correlation	*p*	Adjusted r Correlation	*p*
STEMI	Troponin	0.382	0.026*	0.519	0.008*
	CKMB	0.219	0.221	−0.099	0.639
	NLR	0.387	0.014*	0.276	0.182
	PLR	0.116	0.476	0.484	0.014*
	MLR	0.381	0.015*	0.130	0.534
	BLR	0.109	0.504	0.145	0.490
	ELR	−0.399	0.011*	−0.242	0.245
NSTEMI	Troponin	0.399	0.044*	0.425	0.070
	CKMB	−0.005	0.980	0.446	0.055
	NLR	0.225	0.174	0.364	0.126
	PLR	0.176	0.291	−0.053	0.829
	MLR	0.018	0.915	0.239	0.325
	BLR	0.042	0.804	0.098	0.690
	ELR	−0.152	0.362	−0.134	0.584
UAP	Troponin	0.250	0.168	0.047	0.822
	CKMB	−0.357	0.041*	−0.067	0.751
	NLR	0.146	0.389	0.527	0.007*
	PLR	0.158	0.352	0.558	0.004*
	MLR	0.276	0.098	0.473	0.017*
	BLR	0.087	0.607	0.445	0.026*
	ELR	0.023	0.891	−0.205	0.325
ACS (STEMI + NSTEMI + UAP)	Troponin	0.465	<0.001*	0.387	<0.001*
	CKMB	0.122	0.239	0.199	0.074
	NLR	0.328	<0.001*	0.279	0.012*
	PLR	0.131	0.164	0.276	0.013*
	MLR	0.217	0.020*	0.250	0.024*
	BLR	0.122	0.195	0.237	0.033*
	ELR	−0.297	0.001*	−0.344	0.002*

r, correlation coefficient; *p*, significance; NLR, neutrophil to lymphocyte ratio; PLR, platelet to lymphocyte ratio; MLR, monocyte to lymphocyte ratio; BLR, basophil to lymphocyte ratio; ELR, eosinophil to lymphocyte ratio.

**Table 3:** AUC, 95 CI, cut-off value, sensitivity, and specificity for Troponin, CKMB, NLR, PLR, MLR, BLR, and ELR in high-risk GRACE Score.

State Variable	Parameter	AUC	95% CI	Cut-off Value	Sensitivity (%)	Specificity (%)	*p*
STEMI	Troponin	0.853	0.712–0.994	5193.6000	0.727	0.714	0.001*
	CKMB	0.714	0.524–0.904	116.0500	0.636	0.667	0.050*
	NLR	0.723	0.548–0.898	8.1298	0.727	0.714	0.041*
	PLR	0.498	0.260–0.736	149.3563	0.545	0.571	0.984
	MLR	0.818	0.648–0.988	0.4735	0.727	0.762	0.004*
	BLR	0.554	0.330–0.778	0.0208	0.545	0.571	0.620
	ELR	0.234	0.068–0.400	0.0170	0.273	0.333	0.015*
NSTEMI	Troponin	0.624	0.385–0.863	191.4500	0.571	0.579	0.340
	CKMB	0.564	0.285–0.843	28.0500	0.571	0.526	0.623
	NLR	0.609	0.398–0.820	4.8642	0.714	0.579	0.402
	PLR	0.571	0.354–0.789	137.7302	0.571	0.474	0.583
	MLR	0.579	0.330–0.828	0.3751	0.714	0.579	0.544
	BLR	0.398	0.133–0.664	0.0228	0.429	0.421	0.435
	ELR	0.128	0.137–0.637	0.0409	0.429	0.421	0.386
UAP	Troponin	0.694	0.458–0.931	31.6000	0.750	0.741	0.216
	CKMB	0.231	0.001–0.462	20.0500	0.250	0.407	0.087
	NLR	0.611	0.222–1.000	3.2967	0.500	0.519	0.480
	PLR	0.704	0.372–1.000	169.7360	0.750	0.667	0.195
	MLR	0.602	0.208–0.996	0.3088	0.500	0.519	0.517
	BLR	0.426	0.066–0.786	0.0175	0.250	0.407	0.637
	ELR	0.398	0.112–0.685	0.0867	0.500	0.519	0.517
ACS (STEMI + NSTEMI + UAP)	Troponin	0.742	0.620–0.864	234.2000	0.636	0.642	0.001*
	CKMB	0.623	0.463–0.782	39.4500	0.591	0.627	0.085
	NLR	0.703	0.584–0.821	6.2256	0.636	0.672	0.004*
	PLR	0.555	0.412–0.698	149.3563	0.545	0.567	0.438
	MLR	0.701	0.558–0.824	0.4144	0.682	0.687	0.007*
	BLR	0.485	0.334–0.636	0.0195	0.500	0.493	0.834
	ELR	0.289	0.163–0.416	0.0359	0.363	0.343	0.003*

AUC, area under the curve; CI, confidence interval; *p*, significance; CKMB, creatinine kinase myocardial band; NLR, neutrophil to lymphocyte ratio; PLR, platelet to lymphocyte ratio; MLR, monocyte to lymphocyte ratio; BLR, basophil to lymphocyte ratio; ELR, eosinophil to lymphocyte ratio.

**Table 4:** Risk analysis model of Troponin, CKMB, NLR, PLR, BLR, MLR, and ELR in high-risk ACS mortality by GRACE Score.

Blood Markers	Univariate		Bivariate	*p*	Multivariate	*p*
		
	High Risk (%)	Low Risk (%)	OR (95% CI)		Adjusted OR (95% CI)	
**Troponin**			2.787 (1.032–7.524)	0.035*	2.049 (1.802–8.218)	0.014*
≥234.2	14 (34.1)	27 (65.9)				
<234.2	8 (15.7)	43 (84.3)				
**CKMB**			2.389 (0.939–6.077)	0.053	1.222 (0.275–5.427)	0.793
≥39.45	15 (35.7)	27 (64.3)				
<39.45	10 (18.9)	43 (81.1)				
**NLR**			3.287 (1.340–8.059)	0.007*	1.652 (1.306–7.753)	0.030*
≥6.2256	17 (36.2)	30 (63.8)				
<6.2256	10 (14.7)	58 (85.3)				
**PLR**			1.484 (0.625–3.527)	0.249	0.971 (0.246–3.835)	0.967
≥149.36	14 (27.5)	37 (72.5)				
<149.36	13 (20.3)	51 (79.7)				
MLR			4.156 (1.634–10.569)	0.002*	4.067 (1.182–13.987)	0.026*
≥0.41	19 (37.3)	32 (62.7)				
<0.41	8 (12.5)	56 (87.5)				
**BLR**			1.433 (0.602–3.410)	0.276	0.610 (0.177–2.098)	0.432
≥0.019	15 (26.8)	41 (73.2)				
<0.019	12 (20.3)	47 (79.7)				
**ELR**			0.370 (0.152–0.903)	0.023*	0.388 (0.111–1.361)	0.139
≥0.035	10 (15.6)	54 (84.4)				
<0.035	17 (33.3)	34 (66.7)				

CI, confidence interval; OR, odds ratio; p, significance; CKMB, Creatinine Kinase Myocardial Band; NLR, neutrophil to lymphocyte ratio; PLR, platelet to lymphocyte ratio; MLR, monocyte to lymphocyte ratio; BLR, basophil to lymphocyte ratio; ELR, eosinophil to lymphocyte ratio.

**Table 5:** Separate two sub-groups outcomes by means of cut-off values.

Events	Troponin		NLR		MLR	
		
	High	Low	High	Low	High	Low
**Age**	OR: 1.455 (0.630–3.356)		OR: 2.083* (1.019–4.449)		OR: 2.148 (1.011–4.565)	
≥65	19	19	25	24	27	22
<65	22	32	22	44	24	42
**Admission Mortality**	OR: 1.013 (0.991–3.090)		OR: 1.808 (0.567–5.772)		OR: 3.214 (0.928–11.130)	
Died	5	6	7	6	9	4
Survive	37	45	40	62	42	60
**GRACE Score**	OR: 2.787* (1.032 – 7.524)		OR: 3.287* (1.340 – 8.059)		OR: 4.156* (1.634– 10.569)	
≥140	14	9	17	10	19	8
<140	27	43	30	58	32	56
**WBC**	OR: 2.719* (1.140 – 6.482)		OR: 5.039* (2.113 – 12.020)		OR: 2.255* (1.037–4.902)	
≥10,000	29	24	38 31	31	36	33
<10,000	12 27	27	9 37	37	15	31
**PLT**	OR: 1.342* (1.041 – 2.979)		OR: 3.000 (0.264–34.096)		OR: 2.583 (0.227–29.339)	
≥120,000	1	1	2	1	2	2
>120,000	38	51	44	66	48	48

OR, odds ratio; p, significance; NLR, neutrophil to lymphocyte ratio; MLR, monocyte to lymphocyte ratio; WBC, white blood cells; PLT, platelet; GRACE, global registry of acute coronary events.

## Data Availability

The data sets used and/or analyzed during the current study are available from the corresponding author upon reasonable request.
